# Phylogenetic Structure and Metabolic Properties of Microbial Communities in Arsenic-Rich Waters of Geothermal Origin

**DOI:** 10.3389/fmicb.2017.02468

**Published:** 2017-12-12

**Authors:** Simona Crognale, Sarah Zecchin, Stefano Amalfitano, Stefano Fazi, Barbara Casentini, Anna Corsini, Lucia Cavalca, Simona Rossetti

**Affiliations:** ^1^Water Research Institute, National Research Council of Italy (IRSA – CNR), Rome, Italy; ^2^Dipartimento di Scienze per gli Alimenti, la Nutrizione e l’Ambiente (DeFENS), Università degli Studi di Milano, Milan, Italy

**Keywords:** microbiome, thermal waters, detoxification processes, arsenic-related genes, arsenite, arsenate

## Abstract

Arsenic (As) is a toxic element released in aquatic environments by geogenic processes or anthropic activities. To counteract its toxicity, several microorganisms have developed mechanisms to tolerate and utilize it for respiratory metabolism. However, still little is known about identity and physiological properties of microorganisms exposed to natural high levels of As and the role they play in As transformation and mobilization processes. This work aims to explore the phylogenetic composition and functional properties of aquatic microbial communities in As-rich freshwater environments of geothermal origin and to elucidate the key microbial functional groups that directly or indirectly may influence As-transformations across a natural range of geogenic arsenic contamination. Distinct bacterial communities in terms of composition and metabolisms were found. Members of *Proteobacteria*, affiliated to *Alpha*- and *Betaproteobacteria* were mainly retrieved in groundwaters and surface waters, whereas *Gammaproteobacteria* were the main component in thermal waters. Most of the OTUs from thermal waters were only distantly related to 16S rRNA gene sequences of known taxa, indicating the occurrence of bacterial biodiversity so far unexplored. Nitrate and sulfate reduction and heterotrophic As(III)-oxidization were found as main metabolic traits of the microbial cultivable fraction in such environments. No growth of autotrophic As(III)-oxidizers, autotrophic and heterotrophic As(V)-reducers, Fe-reducers and oxidizers, Mn-reducers and sulfide oxidizers was observed. The *ars* genes, involved in As(V) detoxifying reduction, were found in all samples whereas *aioA* [As(III) oxidase] and *arrA* genes [As(V) respiratory reductase] were not found. Overall, we found that As detoxification processes prevailed over As metabolic processes, concomitantly with the intriguing occurrence of novel thermophiles able to tolerate high levels of As.

## Introduction

In aquatic environments, arsenic (As) can be found in four oxidation states (+V, +III, 0, -III) showing various levels of toxicity and bioavailability (Rahman and Hassler, 2014). The speciation of As is largely controlled by pH and redox potential (Vu et al., 2003). Arsenate [As(V)] is the predominant form of inorganic arsenic in aqueous aerobic environments, mainly found as H_2_AsO_4_^-^ at pH < 6.9 and as HAsO_4_^2-^ at higher pH values. Arsenite [As(III)] prevails under reducing conditions, as uncharged H_3_AsO_3_^0^ at pH < 9.2 (Oremland and Stolz, 2003). The occurrence of As in groundwater and surface waters is determined by geothermal inputs and geogenic processes, such as the dissolution of As-bearing minerals in soils and sediments (Smedley and Kinniburgh, 2002; Bissen and Frimmel, 2003; Fendorf et al., 2010).

Microbial activity is linked to the biogeochemistry of arsenic and microorganisms are able to directly catalyze As transformations in natural environments or mediate these processes by coupling with other redox reactions such as nitrate-, sulfate-, manganese-, and iron-reduction (Rittle et al., 1995; Fendorf et al., 2010; Lee, 2013; Zhang et al., 2017). Aquatic microorganisms have evolved different mechanisms to resist to high As concentrations and metabolize it, including sorption, mobilization, precipitation and redox and methylation transformation (Huang, 2014). Microbial resistance to both As(V) and As(III) is based on a detoxification system that reduces As(V) to As(III) through a cytoplasmic arsenate reductase and extrudes the latter from the cellular compartment by means of a membranous As(III) efflux pump (*ars* system) (Silver and Phung, 2005a,b). This resistance mechanism is widespread in nature and present in bacteria, archaea and eukaryotes (Mukhopadhyay et al., 2002; Stolz et al., 2006). As-resistant bacteria were found to mainly belong to *Proteobacteria* (*Alpha*-, *Beta*- and *Gammaproteobacteria*), *Firmicutes* and *Actinobacteria* (Páez-Espino et al., 2009). As(III) oxidation is also a potential detoxification process found in heterotrophic bacteria such as *Herminiimonas arsenicoxydans* (Muller et al., 2006).

Besides resistance mechanisms, arsenic metabolisms associated with cell growth were also described. Many aerobic microorganisms are able to oxidize As(III) to As(V) in the periplasm owing to the presence of an arsenite oxidase (*aio* gene) (Lett et al., 2012). The *aio* system is phylogenetically and ecologically widespread in bacteria and archaea (van Lis et al., 2013).

Occasionally, As(III) oxidation may be combined with nitrate respiration or integrated into electron transport chain of anoxygenic photosynthesis (Hoeft et al., 2007; Zargar et al., 2012). This anaerobic pathway is catalyzed by the *arxAB* system, a new arsenite oxidase firstly discovered in *Alkalilimnicola ehrlichii* strain MLHE-1 (Zargar et al., 2010).

It is well documented that As(III) prevails in geothermal systems, with the rapid oxidation to As(V) occurring in the outflow channels (Wilkie and Hering, 1998; Jackson et al., 2001; Langner et al., 2001; Inskeep et al., 2005). So far, geothermal As-oxidizing bacteria are mainly affiliated to the bacterial phyla *Aquificae*, *Chloroflexi*, *Deinococcus–Thermus*, and to the *Archaea* domain (Sehlin and Lindström, 1992; Gihring and Banfield, 2001; Donahoe-Christiansen et al., 2004; Tang et al., 2011). Differently from geothermal systems, As(III) oxidizers in mesophilic environments include mainly *Alpha*-, *Beta-*, and *Gammaproteobacteria* (Stolz et al., 2006; van Lis et al., 2013).

Moreover, many microorganisms are able to perform a dissimilatory As(V) reduction using As(V) as electron acceptor and different inorganic (e.g., H_2_) and organic compounds (e.g., small organic acids, sugars and complex aromatic substrates like benzoate and toluene) as electron donor (Stolz and Oremland, 1999; Kumari and Jagadevan, 2016). This periplasmic anaerobic respiration of As(V) is driven by a membranous arsenate reductase encoded by *arr* genes (Huang, 2014). As(V) reducers were affiliated to *Crenarchaeota*, *Aquificae*, *Chrysiogenes*, *Deferribacteres*, *Halanaerobacter*, and *Beta*-, *Gamma*-, *Delta*-, *Epsilonproteobacteria* (Stolz et al., 2006). A chemolithotrophic metabolism based on As(V) reduction to As(III) coupled with the oxidation of sulfide to sulfate was shown in strain MLMS-1, anaerobic deltaproteobacterium isolated from an alkaline, hypersaline lake in California (Hoeft et al., 2004). This bacterium was found to be able to grow by disproportionation of monothioarsenates, molecules which contain internally S^2-^ as an electron donor and As(V) as electron acceptor (Planer-Friedrich et al., 2015). Thioarsenates were shown to serve as energy source for *Aquificales* in the hydrothermal systems of Yellowstone National Park (Haertig and Planer-Friedrich, 2012) and to support, as monothioarsenate, the growth of the aerobic hyperthermophilic bacterium *Thermocrinis ruber* strain OC14/7/2 (Haertig et al., 2014) and haloalkaliphilic, anoxygenic photosynthetic purple sulfur bacteria (Edwardson et al., 2014).

Recently, the attention is focused on structural and metabolic characteristics (related to arsenic metabolism and resistance) of mixed microbial communities in As-contaminated environments (Escudero et al., 2013; Andres and Bertin, 2016). These studies suggested that As-related genes might help in detecting, monitoring and managing As contamination of either geogenic or anthropogenic origin. However, still little is known about the phylogenetic composition and functional properties of the aquatic microbial communities involved in As transformation processes. Most of the studies performed so far were mainly focused on the microbial cultivable fraction, without exploiting the potential of the overall community to face As contamination.

It is known that the activities of indigenous bacteria in As-rich aquatic environments may control the biogeochemical cycle of this element by mediating other redox reactions. Arsenic mobility in aquatic environments was shown to be controlled by biogeochemical redox transformations of iron and manganese, especially in alluvial plains where organic matter is readily available for biotic reactions (Borch et al., 2010; Jones et al., 2012). Additionally, the involvement of microorganisms in sulfate reduction may directly affect the precipitation of As sulfides species (i.e., realgar) or the As(III) oxidation in geothermal environments (Burton et al., 2013; Rodriguez-Freire et al., 2016). Nitrate-linked microbial transformation were also shown to play an important ecological role in As-contaminated aquatic environments (Oremland and Stolz, 2003).

Our study aimed to evaluate the structural and phylogenetic patterns in the microbiome of As-rich waters of geothermal origin, as those typically found in the Cimino-Vico volcanic area (Central Italy), and to elucidate the key microbial functional groups that directly or indirectly may influence As-transformations across a natural range of geogenic arsenic contamination.

## Materials and Methods

### Sampling Site

Water samples were collected from the Cimino-Vico volcanic area (Central Italy) (Supplementary Figure S1), a complex hydrogeological system characterized by several perched aquifers and a continuous basal aquifer flowing through volcanites (Baiocchi et al., 2013). In this volcanic region, widespread hydrothermal circulation was reported as underlined by the occurrence of geothermal fields (Dall’aglio et al., 2001; Casentini and Pettine, 2010). The natural occurrence of arsenic is explained by the complexity of the hydrostratigraphy, the structural setting of the area and the related mixing occurring between water circulating in the basal volcanic aquifer and the fluids that rise from depth, all of which characterize the active geothermal system (Angelone et al., 2009; Armiento et al., 2015; Cinti et al., 2015). We selected eight different freshwater sources, differently influenced by the rising of As-rich thermal waters (50–64°C) from a deep aquifer consisting of Mesozoic sedimentary rocks, locally uplifted, fractured, and faulted (Baiocchi et al., 2013). The sampling survey was performed in winter (November, 2015), and samples included (i) thermal waters: one hot spring (SSC) derived from a flowing well 346 m deep and two pools (CAR, PAL) collecting waters from natural hot springs; (ii) groundwaters from four wells (OLI, BEL, ANG, FON); (iii) surface waters sampled 2 m away from the west side shore of the Lake Vico (VICO) directly affected by hydrothermal upwelling.

### Water Chemistry

Temperature (T), pH, electrical conductivity (EC), and dissolved oxygen (DO) were measured on-site by field probes (Hach HQ 40d). Sulfides, iron (total and II) were determined by spectrophotometric methods (Casentini et al., 2016). Water samples (50 ml) were directly stored at 4°C for anion analysis by ion chromatography (Dionex DX-120); 50 ml of water were filtered *in situ* through 0.45 μm cellulose acetate membrane filters (Whatman), acidified with 2% HNO_3_, and stored at 4°C for cation analysis by ICP-MS equipped with Octapole Reaction System (ORS) (Agilent 7500). Arsenic speciation was assessed by hydride generation-absorption spectrometry (HG-AAS, Perkin Elmer AAnalyst 800). Arsenite determination was carried out using HCl 2% as carrier and reduction to arsine gas was performed with NaBH_4_ 0.4% in acetate buffered samples at pH 4–4.5. As_tot_ was analyzed by HG-AAS prior reduction to As(III) by 5% KI/Acid Ascorbic solution. As(V) concentration was obtained by the difference (details in Casentini et al., 2016).

### Prokaryotic Abundance and Community Composition by Flow Cytometry and CARD-FISH

Each water sample was fixed in formaldehyde solution (FA, 1% vol/vol final concentration) and kept at 4°C for a maximum of 24 h. The flow cytometer A50-micro (Apogee Flow System, Hertfordshire, England), equipped with a solid state laser set at 20 mV and tuned to an excitation wave length of 488 nm, was used to characterize microbial communities in fixed samples. The volumetric absolute cell counting was carried out on samples stained with SYBR Green I (1:10,000 dilution; Molecular Probes, Invitrogen). Apogee Histogram Software (v89.0) was used to plot and analyze data; the light scattering signals (forward and side scatters) and the green fluorescence (530/30 nm) were considered for the single cell characterization. Thresholding was set on the green channel and voltages were adjusted to place the background and instrumental noise below the first decade of green fluorescence. Samples were run at low flow rates to keep the number of events below 1000 events s^-1^. The intensity of green fluorescence emitted by SYBR-positive cells allowed for the discrimination among cell groups exhibiting two different nucleic acid content (cells with Low Nucleic Acid content - LNA; cells with High Nucleic Acid content - HNA) (Amalfitano et al., 2014). The forward scatter signals (FSC expressed in absolute units, AU) can be considered to be relatively proportional to cell size (Tzur et al., 2011), and related to the size variations of LNA and HNA cells.

Further fixed samples (10–200 mL depending on total cell abundance) were filtered on polycarbonate membrane filters (pore size 0.2 μm, 47 mm diameter, Nuclepore) by gentle vacuum (<0.2 bar) then washed with 20 ml of Milli-Q water. The filters were stored in Petri dishes at -20°C until further processing. CARD-FISH analysis was performed following the protocol optimized by Fazi et al. (2007, 2013) using specific rRNA-target HRP-labeled probes (Biomers, Ulm, Germany): EUB338 I-III for *Bacteria*; ALF968 for *Alphaproteobacteria*; BET42a for *Betaproteobacteria*; GAM42a for *Gammaproteobacteria*; DELTA495 for *Deltaproteobacteria*; CFX and GNSB for *Chloroflexi*, LGC354mix for *Firmicutes*, CF319a for *Flavobacteria*, PLA46 for *Planctomycetes*, TM7905 for TM7, HGC69A for *Actinobacteria* and ARCH915 for *Archaea*. Details of probes are available at probeBase (Greuter et al., 2016). The stained filter sections were inspected on a Leica DM LB30 epifluorescence microscope (Leica Microsystems GmbH, Wetzlar, Germany) at 1000X magnification. At least 300 cells were counted in > 10 microscopic fields randomly selected across the filter sections. The relative abundance of hybridized cells was estimated as the ratio of hybridized cells to total DAPI-stained cells.

### DNA Extraction

For DNA extraction and subsequent PCR applications water samples were filtered (750–1000 ml) through polycarbonate membranes (pore size 0.2 μm, 47 mm diameter, Nuclepore) and immediately stored at -20°C. DNA extraction was performed with PowerSoil^®^ DNA Isolation Kit (MoBio – Carlsbad, CA, United States) by following the manufacturer’s instructions. The quality of extracted DNA (1.6 < A_260/280_ < 1.8 and A_260/230_ > 2) was analyzed with a Nanodrop 3300 (Thermo Scientific, *Italy*). DNA was stored at -20°C in small aliquots.

### Bacterial 16S rRNA Gene Pyrosequencing

Bacterial 16S rRNA genes was amplified in triplicate from DNA samples using primers 27F (5′-GAG AGT TTG ATC CTG GTC CAG-3′) and 1495R (5′-CTA CGG CTA CCT TGT TAC GA-3′). Reactions were set up in 25 μL volumes containing 10 ng of DNA, 0.3 μM primers and 1x Taq PCR MAstermix kit (QIAGEN, Hilden, Germany). The thermal protocol included 5 min of denaturation at 95°C followed by 35 cycles of denaturation at 95°C for 1 min, 40 s annealing at 55°C and 1 min and 40 s elongation at 72°C; the final elongation was performed at 72°C for 10 min. Replicated amplicons from the same sample were pooled and purified using the MinElute PCR Purification Kit (QIAGEN) to reach a final concentration of 20 ng μL^-1^. Pyrosequencing was performed with primer 27F at Molecular Research LP (MRDNA, Shallowater, TX, United States) by bacterial Tag-Encoded FLX Amplicon Pyrosequencing (bTEFAP).

The sequences were processed and analyzed using the QIIME software tools (Caporaso et al., 2010b). Sequences shorter than 200 bp, with barcodes or primer biases, homopolymers and chimeras were filtered out from the dataset. High quality sequences selected for the analysis were trimmed at 400 bp and grouped into Operational Taxonomic Units (OTUs) with 97% similarity with the uclust method (Edgar, 2010) according to the last SILVA SSU Ref database (Quast et al., 2013). Representative sequences for each OTU were selected and aligned using the PyNAST algorithm (Caporaso et al., 2010a), taxonomy was assigned and OTU tables were generated for each sample.

### Phylogenetic Analysis

The phylogeny of the majority of reads retrieved from thermal samples (SCC, CAR, PAL) was not successfully assigned with QIIME. Therefore, these sequences were separately compared to the GenBank database with BLASTn. Together with the closest relatives, the sequences were aligned on the MEGA software version 6 (Tamura et al., 2013) using MUSCLE (Edgar, 2004) and the phylogenetic tree was calculated with the Maximum Likelihood method based on the Tamura-Nei model (Tamura and Nei, 1993).

### Real-Time Quantification of Arsenic-Related Functional Genes

The screening of functional genes known to be involved in the arsenic cycle was performed by PCR using the Taq98^TM^ Hot Start 2X Master Mix (Lucigen, United States) according to the manufacturer’s instructions. 10 different primer sets were used for the amplification of *aioA*, *arrA*, *arsB* and *arsC* according to the protocols reported in literature (Supplementary Table S1). In detail, arsenite oxidase gene (*aioA*) was targeted using aroA#1F - aroA#1R primers according to Karn and Pan (2016); arrAf – arrAr primer set (Malasarn et al., 2004) was used for the amplification of arsenate respiratory reductase gene (*arrA*). Arsenate cytoplasmic reductase (*arsC*) was amplified using amlt-42-F/amlt-376-R primers according to Sun et al. (2004). Quantification of arsenite transporter (*arsB*) was carried out using arsB#1F - arsB#1R primers according to Achour et al. (2007). qPCR reactions were performed using Sso Advanced Universal SYBR Green Supermix (BIO-RAD, United States) according to the manufacturer’s instructions on a CFX96 Touch Real-time PCR detection system. Melting curves were performed for each reaction to confirm the purity of amplified products.

### Most Probable Number

Most Probable Number (MPN) counts were determined with triplicate 10-fold dilution series (to 10^-9^) using liquid selective growth media, inoculated and incubated for 30 days at the same temperature of the original water sample. In particular, MPN was used to quantify aerobic heterotrophs, autotrophic and heterotrophic As(III)-oxidizers, As(V)-reducers, sulfide-oxidizers, sulfate-reducers, nitrate-reducers, iron-oxidizers, iron-reducers and manganese-reducers. For autotrophic and heterotrophic As(III)-oxidizers and As(V)-reducers, 1 ml of water sample was added to 9 ml of basal mineral medium (Corsini et al., 2014) supplemented with sodium arsenite (NaAsO_2_) or disodium hydrogen arsenate (Na_2_HAsO_4_) at a final concentration of 100 mg/L with or without sodium lactate (1 g/L final concentration). Selective growth media for heterotrophs, sulfide-oxidizers and sulfate-reducers were prepared according to Cote and Gherna (1994). Growth media suggested by Lovley (2006) were used to quantify nitrate-, iron- and manganese-reducers with a mix of carbon sources at final concentration of 1g/L (sodium acetate, glucose and lactate in 2:1:1 ratio). The enumeration of iron-oxidizers was performed by using a FeSO_4_-based mineral medium as described in Barron and Lueking (1990). The growth of As(V)-, Fe-, sulfate-, nitrate- and Mn-reducing bacteria was estimated under anaerobic conditions carefully controlled during either the sampling and laboratory incubation. In particular, samples were dispensed into culture tubes, sealed under N_2_ atmosphere with butyl rubber stoppers. The reducing growth media were autoclaved and, before inoculation, again stripped with nitrogen under axenic conditions.

MPN (cell/mL) was estimated as described in Sutton (2010). Positive tubes were analyzed at the end of the incubation to confirm the screened metabolisms by evaluating the occurrence of the expected end-products.

### Statistical Analysis

The non-parametric Mann–Whitney *U*-test was applied to verify the statistical difference between thermal and non-thermal waters for all physical, chemical and microbial parameters (Clarke, 1993). The chemical variables with *p* < 0.05 were incorporated into a Non-metric MultiDimensional Scaling ordination plot (NMDS) in order to graphically synthesize the Euclidean dissimilarity between the two groups of water samples. Chemical and microbial abundance data were then projected onto the NMDS ordination using a vector-fitting procedure, in which the length of the arrow is proportional to the correlation between NMDS-axes and each variable. This method allowed determining the variation pattern of each projected variable discriminating the two groups of waters (Foulquier et al., 2013; Amalfitano et al., 2014). Chemical data were log-transformed, whereas values of cell abundance of the major prokaryotic subgroups, including flow cytometry (LNA and HNA cells) and CARD-FISH data (i.e., major phyla and classes of *Proteobacteria*) was normalized by log(X+1).

A multi-group SIMilarity PERcentage test (SIMPER), performed on NGS data using the Euclidean similarity measure, was run to identify the microbial phylogenetic groups that were primarily responsible for observed differences between thermal waters and groundwaters.

Non-parametric MANOVA (NPMANOVA) was used to test if microbial taxa obtained from 16S rRNA bar-coded pyrosequencing differed statistically among thermal waters and groundwaters.

## Results

### Water Chemistry

**FIGURE 1:**
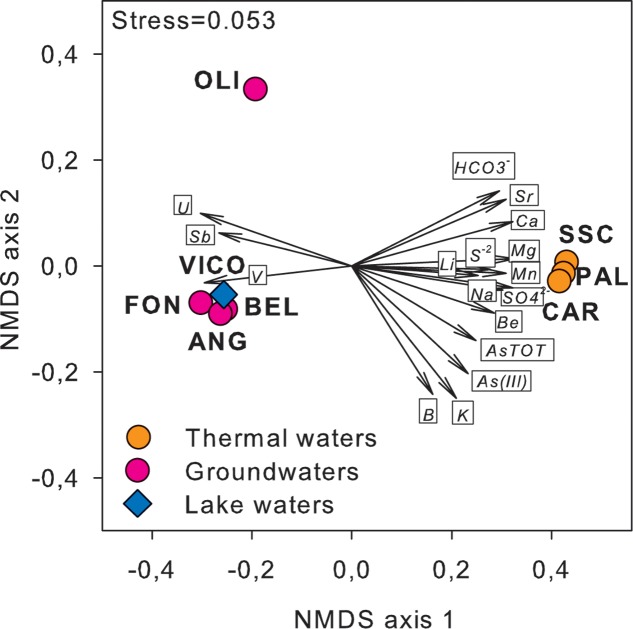
NMDS ordination plot, based on Euclidean distance matrix of log-transformed data, showing the variation patterns of chemical variables that varied significantly between thermal and non-thermal waters. The vector length is proportional to the correlation between the NMDS axes and each chemical variable. The stress value (i.e., <0.2) suggests for an accurate representation of the dissimilarity among water samples.

Major physical and chemical parameters (T, pH, EC, DO, HCO_3_^-^, SO_4_^-2^) differed significantly based on water origin (**Table 1**). Thermal waters showed high EC (4610-5680 μS/cm), high concentrations of SO_4_^-2^ (1264–1670 mg/L), typical of geothermal environments and highest temperature values (55.5–58°C). Groundwaters showed high values of V and U and low values of EC and sulfates. Nitrate concentrations reached the highest value in FON (20 mg/L). Nitrites and phosphates were below detection limits in all samples. Among other ions analyzed (Supplementary Table S2), Sr and Li showed highest values in thermal waters (14270 and 176 μg/L respectively) whereas B varied between 73 (groundwater) and 1206 μg/L (thermal waters). The concentrations of Fe and Mn were generally low and ranged between 0.15 and 0.84 mg Fe/L and 0.3 and 27 μg Mn/L. The highest As concentration was found in thermal waters (329.2–362.1 μg/L). Total arsenic concentrations showed relatively lower concentrations in VICO surface waters (20.9 μg/L) and groundwaters (22.9–182.4 μg/L). As(III) was more concentrated in PAL, CAR and VICO than in groundwaters; the geothermal sample SSC showed less than 4% of As(III). The NMDS ordination allowed visualizing the chemical dissimilarity between thermal and non-thermal waters, also showing the variation pattern of those variables that varied significantly between the two groups of waters (**Figure 1**).

**Table 1:** Main biogeochemical parameters of thermal waters, groundwaters and lake waters.

Samples	T (°C)	pH	EC (μS/cm)	DO (mg/l)	Fe (mg/L)	Mn (μg/L)	U (μg/L)	V (μg/L)	HCO_3_^-^ (mg/L)	S^-2^ (mg/L)	F^-^ (mg/L)	Cl^-^ (mg/L)	NO_3_^-^ (mg/L)	SO_4_^-2^ (mg/L)	As_tot_ (μg/L)	As(III) (μg/L)	% As(III)
PAL	58.0	6.44	5680	0.2	0.38	23.2	0.3	0.5	1160	1.5	3.0	12.9	1.3	1554.8	362.1	107.4	29.7%
SSC	57.8	6.41	5660	0.2	0.27	16.7	0.1	0.5	1121	2.1	2.8	12.6	<LOD	1670.4	351.9	12.0	3.4%
CAR	55.5	6.49	4610	0.2	0.67	27.0	0.2	0.3	1026	0.1	3.2	13.5	<LOD	1263.9	329.2	172.5	52.4%
OLI	24.9	6.65	724	1.33	0.54	2.5	8.1	3.4	708	0	3.0	15.3	6.4	20.0	22.9	0.0	0.0%
BEL	21.7	7.04	356	3.16	0.84	7.1	1.2	6.1	232	0	4.0	19.6	5.9	33.6	152.1	5.4	3.6%
ANG	18.7	6.28	250	7.21	0.78	3.3	2.1	13.2	195	0	1.5	9.1	3.2	15.8	182.4	8.9	4.9%
FON	18.2	6.69	269	7.95	0.15	0.3	5.5	16.0	122	0	1.8	19.2	20.0	21.3	51.9	1.0	1.9%
VICO	14.0	8.28	352	9.06	0.61	1.8	3.7	1.4	244	0	1.0	16.8	1.1	81.8	20.9	7.4	35.4%


*The complete dataset is reported in Supplementary Table S2. LOD, limit of detection.*

### Prokaryotic Cell Abundance and Community Structure

Prokaryotic abundance was higher in lake waters (2.3 × 10^6^ ± 1.9 × 10^4^ cell/ml) and thermal waters (between 5.1 × 10^4^ ± 9.3 × 10^1^ cell/ml and 6 × 10^5^ ± 2.5 × 10^3^ cell/ml) than in groundwaters (between 2.2 × 10^4^ ± 2.6 × 10^2^ cell/ml and 3.3 × 10^5^ ± 4.7 × 10^3^ cell/ml) (**Table 2**). As assessed by flow cytometry, the ratio between the mean green fluorescence and forward scatter signal intensity of prokaryotes was lower in thermal waters than in non-thermal waters. In groundwaters and surface waters, HNA cells showed lower percentages than those found in thermal waters (26% in lake water and 35.2% in groundwater except BEL with HNA = 87%). On average, the mean fluorescence intensity of the HNA cells was approximately four times higher than that of LNA cells.

**Table 2:** Main cytometric characteristics of thermal waters, groundwaters and lake waters.

	Total prokaryotes			LNA cells (%)	HNA cells (%)
			
	PAB 10^4^ cells/ml	Mean fluo Green FU	Mean FSC FU		
PAL	5.1	239	93	6.1	93.9
SSC	11.3	101	34	50.0	50.0
CAR	60.0	315	194	14.7	85.3
OLI	6.3	78	29	70.5	29.5
BEL	33.3	180	44	13.0	87.0
ANG	3.4	96	29	53.9	46.1
FON	2.2	80	21	70.0	30.1
VICO	231.0	67	19	74.1	25.9


*PAB, prokaryotic abundance. FSC, forward side scatter. LNA, low nucleic acid content cells. HNA, high nucleic acid content cells.*

**FIGURE 2:**
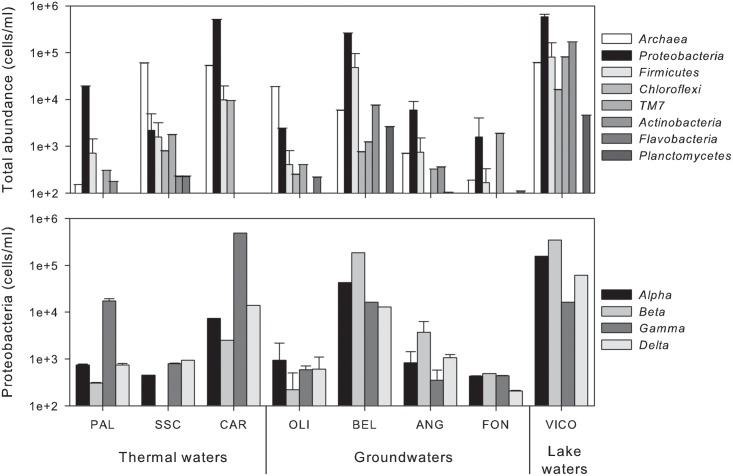
Abundance of phylogenetic clusters (*Archaea* and main phyla within *Bacteria*) **(upper)** and single classes within *Proteobacteria* **(lower)** in thermal waters, groundwaters and lake waters.

As shown in **Figure 2**, the main microbial components belonged to bacteria domain (from 15% up to 95% of total cells) and were mainly affiliated to *Proteobacteria. Alpha*- and *Betaproteobacteria* were the most abundant groups retrieved in groundwaters and surface waters, whereas *Gammaproteobacteria* dominated in thermal waters (reaching 81.5% of bacterial cells in CAR sample). TM7 division was mainly present in groundwaters reaching the highest abundance in FON sample (1.9 × 10^3^ ± 1.4 × 10^2^ cell/mL). *Archaea* were found in all screened samples with abundances ranging between 1.9 × 10^2^ ± 0.2 × 10^1^ cell/mL and 6.0 × 10^4^ ± 1.1 × 10^3^ cell/mL (**Figure 2**).

**FIGURE 3:**
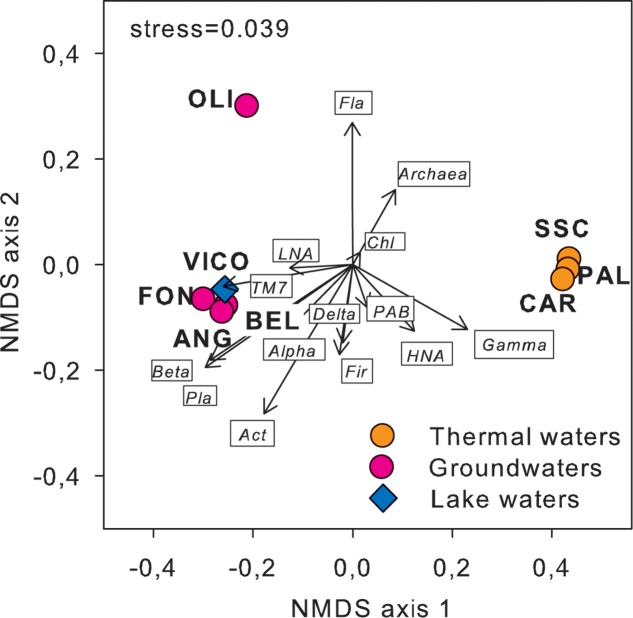
Cell abundance variation of the major prokaryotic subgroups, including flow cytometry (LNA and HNA cells) and CARD-FISH data (i.e., major phyla and classes of *Proteobacteria*), projected onto the NMDS ordination synthesizing the chemical dissimilarity between thermal and non-thermal waters (see **Figure 1**). The vector length is proportional to the correlation between the NMDS axes and each microbial variable, upon normalization by log(X+1). *Act*, *Actinobacteria*; *Chl*, *Chloroflexi*; *Fir*, *Firmicutes*; *Fla*, *Flavobacteria*; *Pla*, *Planctomycetes*; *Alpha*, *Alphaproteobacteria*; *Beta*, *Betaproteobacteria*; *Gamma*, *Gammaproteobacteria*; *Delta*, *Deltaproteobacteria*; LNA, low nucleic acid content cells; HNA, high nucleic acid content cells; PAB, prokaryotic abundance.

Following a vector-fitting procedure onto the NMDS ordination plot based on chemical dissimilarity, we showed that *Archaea*, *Gammaproteobacteria* and HNA cells were relatively more abundant in thermal waters, whereas *Betaproteobacteria*, *Planctomycetes*, TM7, *Alphaproteobacteria*, and LNA cells were more represented in non-thermal waters (**Figure 3**).

### Next Generation Sequencing (NGS)

A total of 71401 ends reads were generated which were assigned into 2988 OTUs. All the resulting 16S rRNA gene fragments were then classified into 24 bacterial phyla, 55 classes, 118 orders, 188 families and 293 genera (Supplementary Table S3). Overall, *Proteobacteria* was the most abundant phylum (46.5% of total OTUs), followed by *Cyanobacteria* (12.1%), *Bacteroidetes* (7.6%), *Nitrospirae* (7.3%), *Firmicutes* (1.2%) and *Acidobacteria* (1.0%). The other 18 phyla represented less than 1.0% of total OTUs. On average, 22.5% of the OTUs showed a very low 16S rDNA identity with known bacterial taxa, with the highest percentage in thermal waters (up to 76.1%).

**FIGURE 4:**
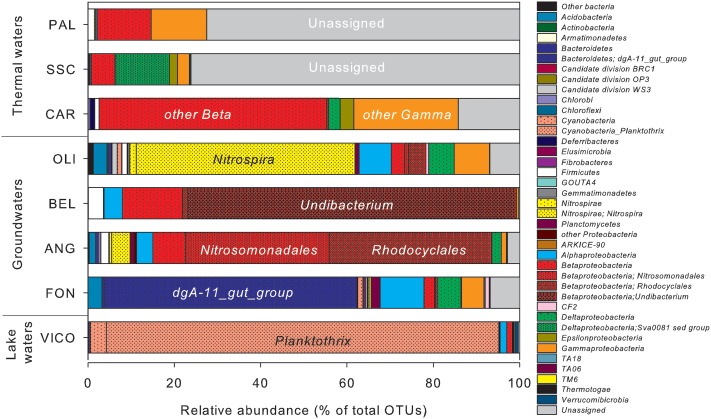
Operational taxonomic units (OTUs) relative abundance in water samples estimated by NGS. Clusters making up less than 1% of total composition were classified as ‘other bacteria’ in thermal waters, groundwaters and lake waters.

The relative abundance of OTUs in water samples differed considerably (**Figure 4**). In surface waters (VICO), *Cyanobacteria* represented the 94.6% of total OTUs and they were mainly affiliated to *Planktothrix* genus (90.9%). *Nitrospirae, Bacteroidetes* and *Proteobacteria* dominated groundwater samples. *Nitrospirae* phylum was largely represented by the genus *Nitrospira* (up to 50.7% of total OTUs). The genus dgA-11 gut group, belonging to *Bacteroidetes*, was found only in groundwaters (up to 58.6% of OTUs in FON sample). *Betaproteobacteria* were mainly represented by orders *Burkholderiales*, *Nitrosomonadales*, and *Rhodocyclales*, affiliated to genera *Undibacterium* and *Azoarcus* and family *Gallionellaceae. Alpha*- and *Gammaproteobacteria* were mainly affiliated to orders *Rhizobiales, Sphingomonadales, Rickettsiales, Rhodospirillales* and to genus *Stenotrophomonas*.

*Proteobacteria* was the predominant phylum reported in thermal waters, mainly affiliated to *Betaproteobacteria* (from 5.3% up to 52.6% of total OTUs) and *Gammaproteobacteria* (from 2.7% up to 24.2% of total OTUs), but the taxonomic affiliation at lower level classification was not identified. *Deltaproteobacteria* were mainly retrieved in one thermal sample (SSC) affiliated to the genus Sva0081_sediment_group, belonging to *Desulfobacterales*.

**FIGURE 5:**
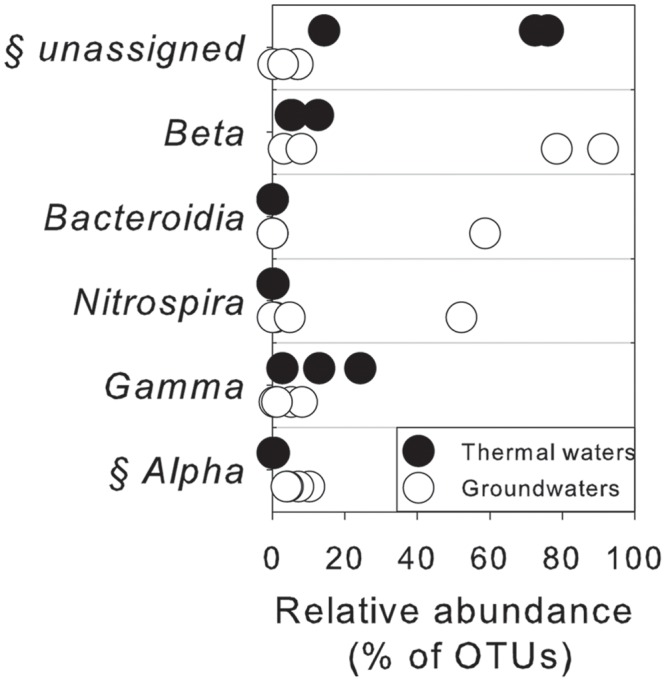
Microbiological variables that more significantly explained the overall dissimilarity (according to SIMPER test) between thermal waters and groundwaters at the class level. §, statistically different between two groups of water according to NPMANOVA analysis (*p* < 0.05).

As assessed by NPMANOVA, thermal waters differed considerably from non-thermal waters. Lake waters were not considered in this analysis, since NGS results highlighted a low diversity in this sample with the predominance of only one genus. According to SIMPER test (Supplementary Table S4), the unassigned portion of OTUs, *Betaproteobacteria*, *Bacteroidia*, *Nitrospira, Gamma-* and *Alphaproteobacteria* were the first six classes that explained more of the overall dissimilarity between the thermal waters and groundwaters, contributing together > 99% of total variability (**Figure 5**).

### Phylogenetic Analysis of Unassigned Bacterial 16S rRNA Gene Sequences from Thermal Waters

**FIGURE 6:**
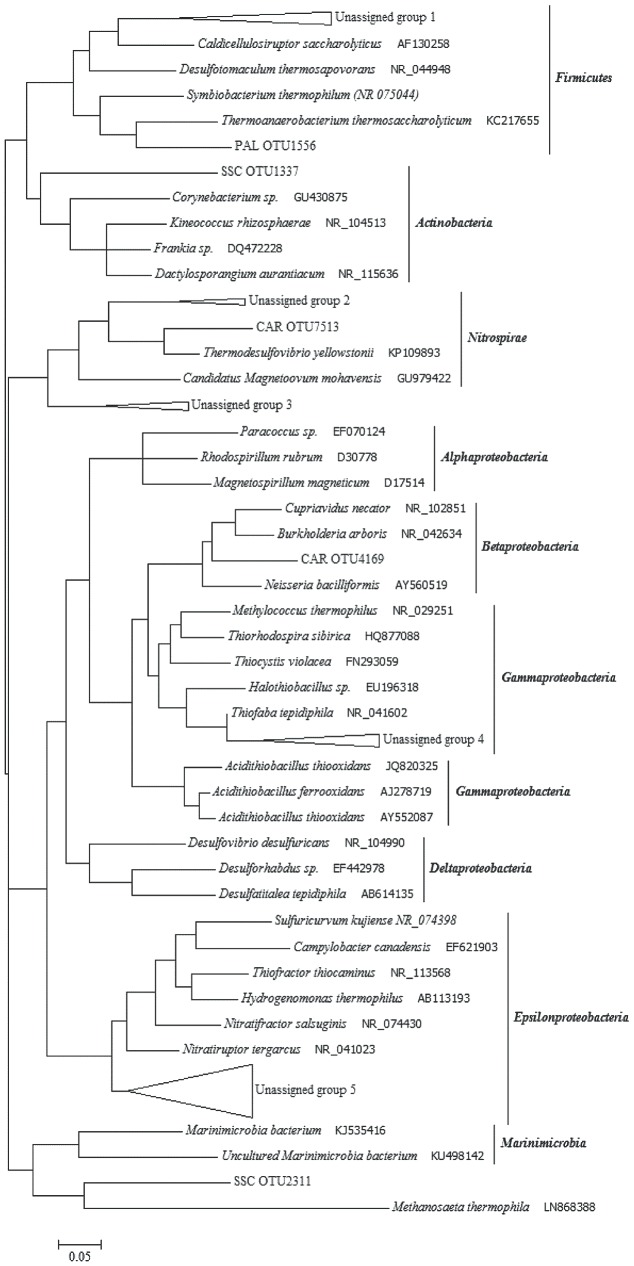
Phylogenetic tree constructed with unassigned 16S rRNA gene sequences obtained from the thermal samples. Unassigned group 1 (76 sequences): PAL OTUs 3865/3142/1624/5721/7240; SSC OTU 5116. Unassigned group 2 (17 sequences): CAR OTUs 1743/886/2900. Unassigned group 3 (13 sequences): SSC OTUs 676/3207/8283/6610; Unassigned group 4 (6 sequences): PAL OTUs 5277/848; SSC OTUs 3302/1374/2608; CAR OTU 5062. Unassigned group 5 (58 sequences): SSC OTUs 1776/2215/8205/6801/8038/363/3362/4634/7530/3704/6025/535/4726/3400/5198/6069/43/7956/1858/3460/6393/6056/2124; PAL OTUs 1583/1883.

A number of sequence reads did not exhibit homology to known microbial taxa, which implies the presence in thermal waters of numerous so far unidentified microorganisms. In SSC and PAL, 76.1 and 72.5% of total OTUs, respectively, were not identified using known databases. In CAR, the unassigned portion was around 14.2% of total OTUs. The phylogenetic analysis of thermal samples showed that most of the unassigned 16S rRNA gene sequences were related to *Epsilonproteobacteria*, *Gammaproteobacteria* and *Firmicutes* (**Figure 6**). Among *Epsilonproteobacteria*, the sequences were related to the members of *Nitratiruptor tergarcus* (∼ 90% of similarity). Additionally, the sequences phylogenetically affiliated to the *Gammaproteobacteria* showed 95% of similarity with *Thiofaba tepidiphila*. The sequences affiliated with *Firmicutes* were only distantly related to *Caldicellulosiruptor saccharolyticus*, *Desulfotomaculum thermosapovorans*, *Thermoanaerobacterium thermosaccharolyticum* and *Symbiobacterium thermophilum* (∼75–80% of similarity). Sequences phylogenetically related to *Actinobacteria*, *Nitrospirae* and *Betaproteobacteria* were also retrieved.

### Most Probable Number

**FIGURE 7:**
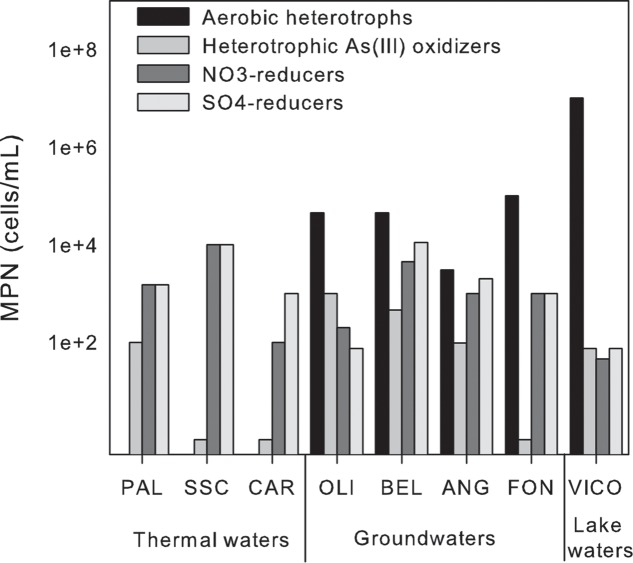
Most probable number (MPN) counts of the main microbial functional groups in thermal waters, groundwaters and lake waters.

Among the tested microbial functions, only nitrate and sulfate reduction, and heterotrophic As(III)-oxidization were found in all the samples (**Figure 7**). In particular, nitrate and sulfate reduction were found as main driving metabolisms in groundwaters where these microbial functional groups were retrieved at high abundance (up to ∼ 10^4^ cells/mL). As expected, lower concentrations were found associated to surface waters in which aerobic heterotrophs were mainly observed. In line with the anoxic reaction environment, no aerobic heterotrophs were found in thermal waters. No growth of autotrophic As(III)-oxidizers, As(V)-reducers, Fe-reducers and oxidizers, Mn-reducers and sulfide oxidizers was observed by MPN. The sole As related metabolism found in the samples was related to heterotrophic As(III) oxidation. The abundance of the latter microbial functional group ranged between 0.1 × 10^1^ and 1 × 10^3^ cell/mL (**Figure 7**).

### Real-Time Quantification of Arsenic-Related Functional Genes

**FIGURE 8:**
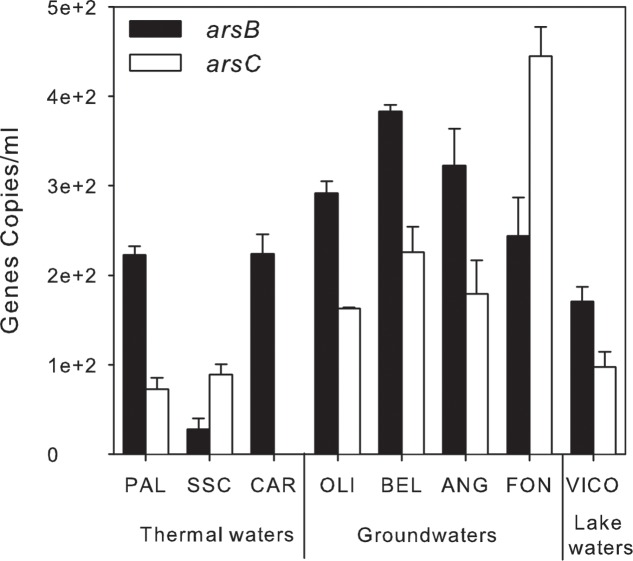
Arsenic-related genes abundance (gene copies/ml) estimated by qPCR in thermal waters, groundwaters and lake waters.

A preliminary screening by PCR showed the occurrence of *arsB*, for arsenite efflux pump, and of cytoplasmic arsenate reductase (*arsC*) genes in all screened samples, whereas arsenite oxidase (*aioA*) and arsenate respiratory reductase (*arrA*) were not amplified with any primer set under any tested condition (Supplementary Table S5). The abundance of *arsB* and *arsC* genes was estimated by real-time PCR. *arsB* and *arsC* genes were found in all samples at low concentration: *arsB* genes ranged between 0.3 × 10^2^± 0.01 × 10^2^ and 0.4 × 10^3^ ± 0.1 × 10^2^ gene copies/ml whereas *arsC* genes were found at concentrations up to 0.4 × 10^3^ ± 0.3 × 10^2^ gene copies/ml except in CAR (<d.l.) (**Figure 8**).

## Discussion

### Arsenic Occurrence and Microbial Community Composition

In this study, As concentrations were in line with previous studies which reported values up to 100 μg/L in groundwaters and 370 μg/L in thermal waters of Cimino-Vico volcanic area (Angelone et al., 2009; Baiocchi et al., 2013; Armiento et al., 2015; Fazi et al., 2016).

In accordance with literature findings reporting a variety of arsenic-resistant and transforming bacterial species in similar environments (Liao et al., 2011; Sarkar et al., 2013; Paul et al., 2015; Bahar et al., 2016), we found the predominance of *Proteobacteria* in groundwaters. In particular, the presence of *Undibacterium* and *Azoarcus* genera in groundwater was often observed in other As-contaminated waters (Griebler and Lueders, 2009; Ghosh and Sar, 2013). Some cultivable representatives of these genera were previously isolated from several environments, including drinking waters, and showed the ability to grow under anoxic condition using nitrate as final electron acceptor (i.e., *Azoarcus* spp.) (Reinhold-Hurek et al., 2015). The high nitrate concentration observed in FON groundwater indicated that the surficial local aquifer could be impacted by agricultural activities and affected by fecal contamination, as suggested by the high relative abundance of OTUs affiliated to dgA-11 gut group (58.6% of total OTUs), usually isolated from animal gastrointestinal tract and feces (Ozutsumi et al., 2005; Tsukinowa et al., 2008).

Consistently with redox conditions commonly found in geothermal environments (Ballantyne and Moore, 1988), thermal waters were generally characterized by low DO content and high As(III) concentration. The prevalence of oxidized As form, observed only in SSC sample, may indicate oxygen exposure during geothermal water uprising.

The high abundance of HNA cells found in thermal waters was in line with the highly selected microbiomes described by NGS. Indeed, LNA and HNA groups detected by flow cytometry are generally considered as constitutive traits of microbial communities in a variety of environments. Their relative contribution was reported to vary according to ecosystem properties and to reflect different phylotype composition, lifestyles and growth potential of the small genome LNA vs. the large genome HNA cells (Bouvier et al., 2007). HNA are also believed as the most active fraction (Lebaron et al., 2002), even though different phylogenetic affiliations were reported (Vila-Costa et al., 2012; Romina Schiaffino et al., 2013). Detection of HNA and LNA has been reported over a wide range of aquatic ecosystems covering large environmental gradients in bacterial (Troussellier et al., 1999; Gasol and del Giorgio, 2000; Andreatta et al., 2004; Thyssen et al., 2005; Sherr et al., 2006) and archaeal populations (Trigui et al., 2011).

In this study, archaea were found at high abundance in thermal waters, likely due to high temperature and reducing redox conditions (Rogers and Amend, 2005; Antranikian et al., 2017). Moreover, in thermal waters, a dominance of OTUs only distantly related to members affiliated to *Epsilonproteobacteria*, *Gammaproteobacteria* and *Firmicutes* was found, thus indicating the occurrence of bacterial biodiversity so far unexplored. Interestingly, the main cluster of unassigned OTUs (group 5 within *Epsilonproteobacteria* in **Figure 6**) was found in SSC sample. Some of the closest relatives are known thermophilic chemolithoautotrophs able to utilize molecular hydrogen as an electron donor and oxygen or nitrate as electron acceptor. Overall, the microbial profiling obtained by NGS analysis revealed in thermal waters the occurrence of microorganisms involved in sulfur and nitrogen biogeochemical cycles (e.g., *Sulfuricurvum kujiense*, *Desulfotomaculum thermosapovorans*, *Thiofaba tepidiphila*, *Nitratiruptor tergarcus*, *Nitratifactor salsuginis, Nitrospira, Azoarcus, Stenotrophomonas*). It is worth to noting that, differently from previous studies performed on high-enthalpy geothermal systems (e.g., Yellowstone National Park), the bacterial phyla typically found in such environments (e.g., *Aquificae*, *Chloroflexi*, *Deinococcus–Thermus*) were not retrieved. This is most likely related to the different geochemical conditions occurring at Cimino-Vico volcanic area (e.g., lower T, nearly neutral pH, slightly reducing redox conditions) which may strongly affect the microbial community structure.

Both As species were found in lake waters and they were likely related to redox processes mediated by high organic carbon availability (litter deposition, surface water runoff, 2.5 mg/L DOC) which represents, together with the light, one of the main driving forces of the complex interplay of biological activities occurring in such environment.

Even though the lake is directly affected by hydrothermal upwelling (Agenzia Regionale per la Protezione Ambientale [ARPA], 2012), the microbiological characteristics of Lake Vico were different from other waters analyzed. The abundance of *Betaproteobacteria* in this aquatic environment showed similar values reported at the same location in previous studies (Davolos and Pietrangeli, 2013; Fazi et al., 2016). Furthermore, the presence of *Planktothrix* was recurrently observed in this area (Manganelli et al., 2016).

### Linking Microbial Metabolic Traits to Arsenic Biogeochemistry

As assessed by the cultivation-dependent approach, sulfate and nitrate reduction and heterotrophic As(III)-oxidation were the main metabolisms occurring in geothermal area. Despite the low concentrations of NO_3_, the involvement of microbial communities in nitrate-reduction was observed in our samples. This suggested that microbial communities could activate this process as a result of an increase in nitrate concentration due to anthropic contamination (e.g., agricultural activity). Nitrate-linked microbial transformation of As was reported in As-contaminated aquatic environments, and some microorganisms were found to mediate anaerobic As(III) oxidation by coupling to nitrate reduction (Zhang et al., 2017). Differently from alluvial environments (Islam et al., 2004), Fe- and Mn-related metabolisms were not found in our samples, since they were not likely to play an essential role in this geothermal area (Piscopo et al., 2006).

Remarkably, As detoxification processes prevail in groundwater and thermal waters. The detection of *arsBC* genes is consistent with previous evidences in a variety of environments (Liao et al., 2011; Davolos and Pietrangeli, 2013; Paul et al., 2015). *arsB* and *arsC* genes were found simultaneously in most of the samples in line with the assumption generally accepted that their environmental distribution is similar (Davolos and Pietrangeli, 2013; Escudero et al., 2013).

Although heterotrophic As(III) oxidation was revealed by MPN analysis, *aioA* gene was not detected by PCR using different primer sets. This finding was probably due to the high microbial diversity in our samples and to the primer coverage that may not be sufficient to capture a high gene diversity. It is known that the investigation of As-related functional genes through PCR approaches as well as through metagenome sequencing is currently difficult in ecological studies (Fahy et al., 2015). A wide variety of *aioA*-like genes exists in natural environments and often these genes show low homology to currently known gene sequences (Hamamura et al., 2009; Jiang et al., 2015). Hence, new primer designs should integrate the most recent sequencing data as well as biochemical data and genetic context (Fahy et al., 2015). The primers in use so far were designed on few microbial strains and were mainly used only on isolates (Inskeep et al., 2007; Quéméneur et al., 2008; Karn and Pan, 2016). Few studies reported the detection of As-related genes in mixed microbial communities in natural environments (Engel et al., 2013; Escudero et al., 2013; Lami et al., 2013), in spite of their presence in different prokaryotic groups such as *Proteobacteria*, *Firmicutes*, *Chrysiogenetes*, *Deinococcus-Thermus*, *Deferribacteres* and *Chrenarchaeota* isolated from a variety of As-rich environments (e.g., mine, arsenical pesticide- or smelter-impacted sites, geothermal sites, geyser, soil and sediments) (Inskeep et al., 2007; Cai et al., 2009; Heinrich-Salmeron et al., 2011; Sultana et al., 2012).

## Conclusion

This study provided the structure and composition of the microbiome exposed to a natural range of geogenic As contamination in geothermal waters. Since the occurrence and distribution of arsenic-related genes were not exhaustively explored in waters of geothermal origin, we provided field insights on the metabolic activities and potentialities of microbial communities related to arsenic detoxification, reduction and oxidation. Remarkably, the microbial communities were able to withstand high As levels, without using it for energetic metabolism. Further studies are needed to investigate the occurrence of novel thermophiles able to tolerate high As concentrations in geothermal environments.

## Author Contributions

SC conducted CARD-FISH, MPN, qPCR experiments and analyzed the whole set of biological data. SA performed FCM measurements, the statistical data analysis and together with SF contributed to the interpretation of the biomolecular data. SZ, AC, and LC performed the bacterial 16S rRNA gene pyrosequencing and the phylogenetic analysis. BC performed the chemical analysis of the water samples and contributed to the interpretation of chemical data. SR conceived and coordinated the study. All authors contributed to the writing of the manuscript.

## Conflict of Interest Statement

The authors declare that the research was conducted in the absence of any commercial or financial relationships that could be construed as a potential conflict of interest.
