# Supplementary material for: Phylogenetic Structure and Metabolic Properties of Microbial Communities in Arsenic-Rich Waters of Geothermal Origin
Source: Front Microbiol. 2017 Dec 12;8:2468. doi: 10.3389/fmicb.2017.02468 (PMC5732945; doi:10.3389/fmicb.2017.02468)
Supplement: Supplementary file 1 [file Table_1.DOCX]

**Table S1**. Primer sets used in this study for PCR amplification. In black bold primers used in qPCR.

| Targeted gene | Primer set and name | | Primer sequence (5’-3’) | Amplicon length (bp) | Reference |
| --- | --- | --- | --- | --- | --- |
| Arsenite oxidase (aioA) | C | aoxBM1-2F | CCACTTCTGCATCGTGGGNTGYGGNTA | 1085 | Quéméneur et al., 2008 |
|  |  | aoxBM3-2R | TGTCGTTGCCCCAGATGADNCCYTTYTC |  |  |
|  |  | aoxBM2-1R | GGAGTTGTAGGCGGGCCKRTTRTGDAT |  |  |
|  | A | aroA #1F | GTSGGBTGYGGMTAYCABGYCTA | 500 | Inskeep et al., 2007 |
|  |  | aroA #1R | TTGTASGCBGGNCGRTTRTGRAT |  |  |
|  | B | aroA #2F | GTCGGYYGYGGMTAYCAYGYYTA | 500 |  |
|  |  | aroA #2R | YTCDGARTTGTAGGCYGGBCG |  |  |
|  | **L** | **aroA #1F** | GTSGGBTGYGGMTAYCABGYCTA | 500 | Karn and Pan 2016 |
|  |  | **aroA #1R** | TTGTASGCBGGNCGRTTRTGRAT |  |  |
|  | D | aroA95f | TGYCABTWCTGCAIYGYIGG | 504 | Hamamura et al., 2008 |
|  |  | aroA599r | TCDGARTTGTASGCIGGICKRTT |  |  |
| Arsenate respiratory reductase (arrA) | **G** | **arrAf** | AAGGTGTATGGAATAAAGCGTTTGTBGGHGAYTT | 160-200 | Malasarn et al., 2004 |
|  |  | **arrAr** | CCTGTGATTTCAGGTGCCCAYTYVGGNGT |  |  |
|  | H | HAArrA-D1f | CCGCTACTACACCGAGGGCWWYTGGGRNTA | 500 | Kulp et al., 2006 |
|  |  | HAArrA-G2R | CGTGCGGTCCTTGAGCTCNWDRTTCCACC |  |  |
|  | I | AS1F | CGAAGTTCGTCCCGATHACNTGG | 625 | Song et al., 2009 |
|  |  | AS1R | GGGGTGCGGTCYTTNARYTC |  |  |
| Arsenate cytoplasmic reductase (arsC) | **E** | **amlt-42-F** | TCGCGTAATACGCTGGAGAT | 334 | Sun et al., 2004 |
|  |  | **amlt-376-R** | ACTTTCTCGCCGTCTTCCTT |  |  |
| Arsenite transporter (arsB) | **F** | **arsB#1F** | GGTGTGGAACATCGTCTGGAAYGCNAC | 750 | Achour et al., 2007 |
|  |  | **arsB#1R** | CAGGCCGTACACCACCAGRTACATNCC |  |  |

**References**:

Achour, A.R., Bauda, P., Billard, P. (2007). Diversity of arsenite transporter genes from arsenic-resistant soil bacteria. Res. Microbiol. 158, 128-137.

Hamamura, N., Macur, R.E., Korf, S., Ackermann, G., Taylor, W.P., Kozubal, M., et al. (2008). Linking microbial oxidation of arsenic with detection and phylogenetic analysis of arsenite oxidase genes in diverse geothermal environments. Environ. Microbiol. doi:10.1111/j.1462-2920.2008.01781.x

Inskeep, W.P., Macur, R.E., Hamamura, N., Warelow, T.P., Ward, S.A., Santini, J.M. (2007). Detection, diversity and expression of aerobic bacterial arsenite oxidase genes. Environ. Microbiol. 9(4), 934–943.

Karn, S.K., Pan, X. (2016). Role of Acinetobacter sp. in arsenite As(III) oxidation and reducing its mobility in soil. Chem. Ecol. 32(5), 460-471.

Kulp, T.R., Hoeft, S.E., Miller, L.G., Saltikov, C., Murphy, J.N., Han, S., et al. (2006). Dissimilatory arsenate and sulfate reduction in sediments of two hypersaline, arsenic-rich soda lakes: Mono and Searles Lakes, California. Appl. Environ. Microbiol. 72(10), 6514–6526.

Malasarn, D., Saltikov, C.W., Cambpell, K.M., Santini, J.M., Hering, J.G., Newman, D.K. (2004). arrA is a reliable marker for As(V) respiration. Science 306, 455.

Quéméneur, M., Heinrich-Salmeron, A., Muller, D., Lièvremont, D., Jauzein, M., Bertin, P.N., et al. (2008). Diversity surveys and evolutionary relationships of *aoxB* Genes in aerobic arsenite-oxidizing bacteria. Appl. Environ. Microbiol. 4567–4573.

Song, B., Chyun, E., Jaffé, P.R., Ward, B.B. (2009). Molecular rmethods to detect and monitor dissimilatory arsenate respiring bacteria (DARB) in sediments. FEMS Microbiol. Ecol. 68, 108–117.

Sun, Y., Polishchuk, E.A., Radoja, U., Cullen, W.R. (2004). Identification and quantification of arsC genes in environmental samples by using real-time PCR. J. Microbiol. Methods 58, 335– 349.
