# Supplementary material for: Phylogenetic Structure and Metabolic Properties of Microbial Communities in Arsenic-Rich Waters of Geothermal Origin
Source: Front Microbiol. 2017 Dec 12;8:2468. doi: 10.3389/fmicb.2017.02468 (PMC5732945; doi:10.3389/fmicb.2017.02468)
Supplement: Supplementary file 2 [file Table_2.DOCX]

**Table S2.** Physical-chemical parameters analyzed

|  | | | | | | | | | | | **As speciation** | | | **ANIONS** | | | | | | |
| --- | --- | --- | --- | --- | --- | --- | --- | --- | --- | --- | --- | --- | --- | --- | --- | --- | --- | --- | --- | --- |
|  | **T**  ***(°C)*** | **pH** | **EC *(µS/cm)*** | **DO**  ***(mg/L)*** | **DO**  ***%*** | **DOC *(mg/L)*** | **HCO_3_^-^*(mg/L)*** | **FeTOT *(mg/L)*** | **FeII *(mg/L)*** | **S^-2^ *(mg/L)*** | **AsTOT *(μg/L)*** | **AsIII *(μg/L)*** | **AsIII/**  ***AsTOT %*** | **F^-^ *(mg/L)*** | **Cl^-^ *(mg/L)*** | **NO_2_^-^ *(mg/L)*** | **NO_3_^-^ *(mg/L)*** | **PO_4_^-^ *(mg/L)*** | **SO_4_^-2^ *(mg/L)*** |  |
| **PAL** | 58 | 6.44 | 5680 | 0.2 | n.d. | 0.9 | 1160 | 0.38 | 0.17 | 1.5 | 362.1 | 107.4 | 29.6% | 3.0 | 12.9 | n.d. | 1.3 | n.d. | 1554.8 |  |
| **SSC** | 57.8 | 6.41 | 5660 | 0.2 | n.d. | 1.3 | 1121 | 0.27 | 0.3 | 2.1 | 351.9 | 12.0 | 3.4% | 2.8 | 12.6 | n.d. | n.d. | n.d. | 1670.4 |  |
| **CAR** | 55.5 | 6.49 | 4610 | 0.2 | n.d. | 0.9 | 1026 | 0.67 | 0.46 | 0.1 | 329.2 | 172.5 | 52.4% | 3.2 | 13.5 | n.d. | n.d. | n.d. | 1263.9 |  |
| **OLI** | 24.9 | 6.65 | 724 | 1.33 | 16.2 | 1.7 | 708 | 0.54 | 0.04 | 0 | 22.9 | 0.0 | 0.0% | 3.0 | 15.3 | n.d. | 6.4 | n.d. | 20.0 |  |
| **BEL** | 21.7 | 7.04 | 356 | 3.16 | 37.8 | 0.6 | 232 | 0.84 | 0.11 | 0 | 152.1 | 5.4 | 3.6% | 4.0 | 19.6 | n.d. | 5.9 | n.d. | 33.6 |  |
| **ANG** | 18.7 | 6.28 | 250 | 7.21 | 82.3 | 0.3 | 195 | 0.78 | 0.18 | 0 | 182.4 | 8.9 | 4.9% | 1.5 | 9.1 | n.d. | 3.2 | n.d. | 15.8 |  |
| **FON** | 18.2 | 6.69 | 269 | 7.95 | 87.2 | 0.3 | 122 | 0.15 | 0.07 | 0 | 51.9 | 1.0 | 2.0% | 1.8 | 19.2 | n.d. | 20.0 | n.d. | 21.3 |  |
| **VICO** | 14 | 8.28 | 352 | 9.06 | 92.88 | 2.5 | 244 | 0.61 | 0.19 | 0 | 20.9 | 7.4 | 35.5% | 1.0 | 16.8 | n.d. | 1.1 | n.d. | 81.8 |  |

|  | **CATIONS** | | | | | | | | | | | | | | | | | | | | | | |  |
| --- | --- | --- | --- | --- | --- | --- | --- | --- | --- | --- | --- | --- | --- | --- | --- | --- | --- | --- | --- | --- | --- | --- | --- | --- |
|  | **Be *(μg/L)*** | **B *(μg/L)*** | **Al *(μg/L)*** | **V *(μg/L)*** | **Cr *(μg/L)*** | **Mn *(μg/L)*** | **Ni *(μg/L)*** | **Cu *(μg/L)*** | **Zn *(μg/L)*** | **Se *(μg/L)*** | **Sr *(μg/L)*** | **Cd *(μg/L)*** | **Sb *(μg/L)*** | **Cs *(μg/L)*** | **Ba *(μg/L)*** | **Hg *(μg/L)*** | **Pb *(μg/L)*** | **U *(μg/L)*** | **Li *(μg/L)*** | **Na *(mg/L)*** | **Mg *(mg/L)*** | **K *(mg/L)*** | **Ca *(mg/L)*** | |
| **PAL** | 2.6 | 1206 | 6.5 | 0.5 | 0.7 | 23.2 | 0.2 | 0.4 | 15.1 | 0.2 | 14270 | <0.1 | 0.2 | 57.2 | 45.0 | < 0.1 | 0.2 | 0.3 | 167 | 36.2 | 165 | 34.7 | 761 | |
| **SSC** | 2.6 | 1183 | 34.0 | 0.5 | 0.7 | 16.7 | 0.3 | 0.3 | 2.1 | 0.1 | 14260 | <0.1 | 0.1 | 55.1 | 37.0 | < 0.1 | 0.1 | 0.1 | 162 | 35.7 | 162 | 34.3 | 702 | |
| **CAR** | 2.5 | 1178 | 2.1 | 0.3 | 0.7 | 27.0 | 0.2 | 0.2 | 0.2 | 0.1 | 12650 | 0.1 | 0.1 | 59.0 | 44.1 | < 0.1 | 0.2 | 0.2 | 176 | 35.6 | 127 | 36.0 | 556 | |
| **OLI** | 0.3 | 73 | 14.0 | 3.4 | 0.8 | 2.5 | 0.6 | 4.4 | 23.0 | 3.0 | 3614 | <0.1 | 0.9 | 85.0 | 268.0 | < 0.1 | 0.3 | 8.1 | 55.8 | 21.4 | 12.2 | 10.6 | 106 | |
| **BEL** | 0.6 | 582 | 10.1 | 6.1 | 0.7 | 7.1 | 0.4 | 0.2 | 5.4 | 0.1 | 295 | < 0.1 | 0.4 | 11.0 | 19.0 | < 0.1 | 0.2 | 1.2 | 128 | 27.3 | 4.6 | 26.9 | 26.7 | |
| **ANG** | 1.1 | 1127 | 70.9 | 13.2 | 0.8 | 3.3 | 0.3 | 0.2 | 594.0 | 0.2 | 288 | <0.1 | 0.9 | 4.1 | 1.8 | < 0.1 | 0.2 | 2.1 | 82.9 | 22.5 | 9.2 | 26.8 | 17.6 | |
| **FON** | 0.5 | 215 | 3.8 | 16.0 | 0.7 | 0.3 | 0.2 | 1.3 | 8.5 | 0.1 | 192 | < 0.1 | 1.4 | 5.6 | 7.6 | < 0.1 | 0.2 | 5.5 | 24.2 | 17.8 | 5.9 | 19.4 | 16.7 | |
| **VICO** | 0.2 | 1176 | 336.0 | 1.4 | 0.7 | 1.8 | 0.2 | 1.7 | 20.0 | 0.2 | 373 | <0.1 | 0.2 | 2.4 | 11.0 | < 0.1 | 0.2 | 3.7 | 25.2 | 24.1 | 14.7 | 25.5 | 25.9 | |
