# Supplementary material for: Phylogenetic Structure and Metabolic Properties of Microbial Communities in Arsenic-Rich Waters of Geothermal Origin
Source: Front Microbiol. 2017 Dec 12;8:2468. doi: 10.3389/fmicb.2017.02468 (PMC5732945; doi:10.3389/fmicb.2017.02468)
Supplement: Supplementary file 5 [file Table_5.DOCX]

**Table S5.** Occurrence of arsenic-resistance genes (aioA. arrA. arsC. arsB) using ten different PCR primer sets (A.B.C.D.L.G.H.I.E.F). - = no amplification observed; + = amplification observed.

|  | **aioA** | | | | | **arrA** | | | **arsC** | **arsB** |
| --- | --- | --- | --- | --- | --- | --- | --- | --- | --- | --- |
|  | A | B | C | D | L | G | H | I | E | F |
| **PAL** | - | - | - | - | - | - | - | - | + | + |
| **SSC** | - | - | - | - | - | - | - | - | + | + |
| **CAR** | - | - | - | - | - | - | - | - | + | + |
| **OLI** | - | - | - | - | - | - | - | - | + | + |
| **BEL** | - | - | - | - | - | - | - | - | + | + |
| **ANG** | - | - | - | - | - | - | - | - | + | + |
| **FON** | - | - | - | - | - | - | - | - | + | + |
| **VICO** | - | - | - | - | - | - | - | - | + | + |
