# Supplementary figures and images for: Phylogenetic Structure and Metabolic Properties of Microbial Communities in Arsenic-Rich Waters of Geothermal Origin
Source: Front Microbiol. 2017 Dec 12;8:2468. doi: 10.3389/fmicb.2017.02468 (PMC5732945; doi:10.3389/fmicb.2017.02468)

Figure S1. Study area and sampling sites

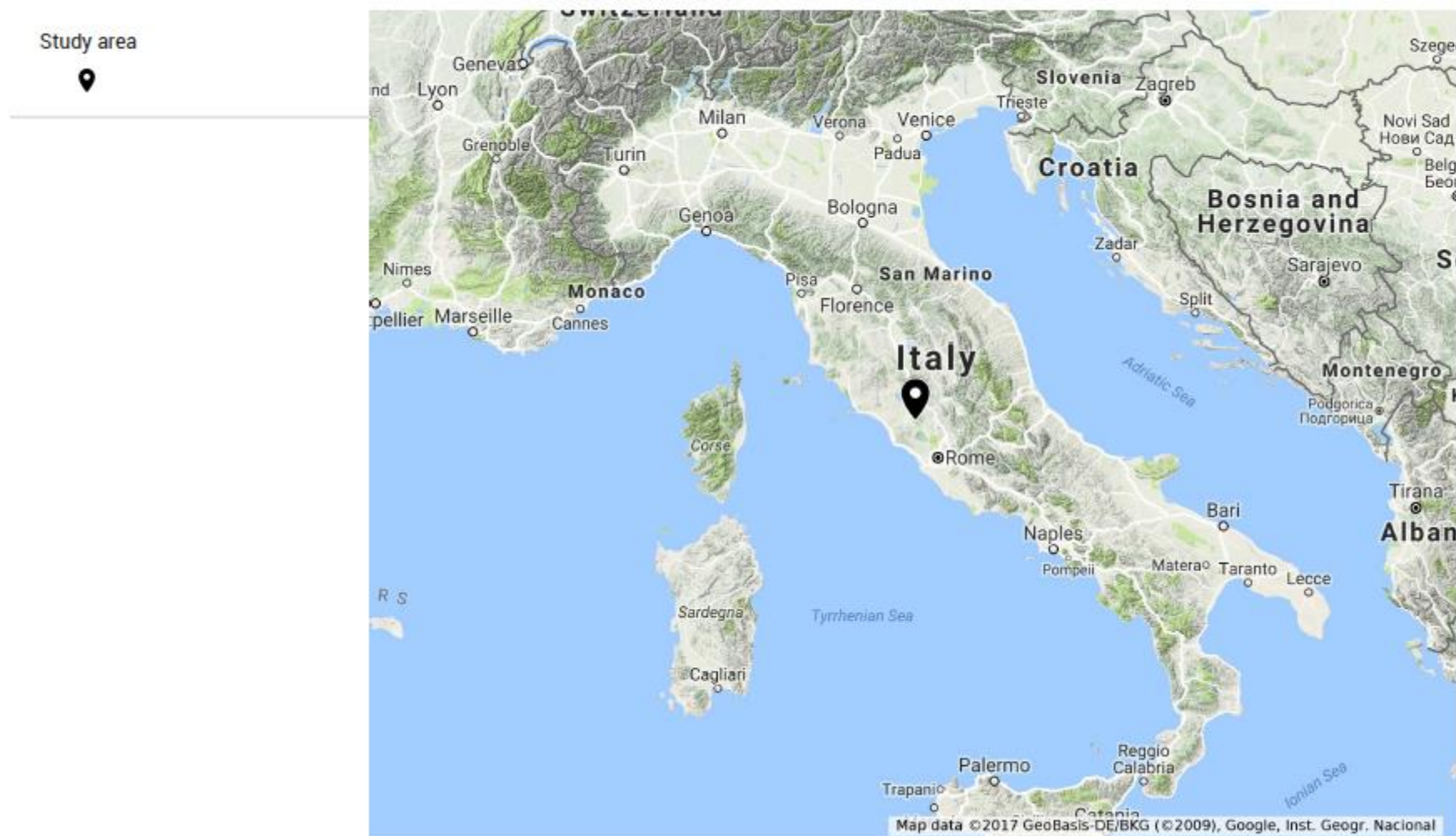

# Sampling sites

- PAL
- SSC
- CAR
- OLI
- BEL
- ANG
- FON
- ◆ VICO

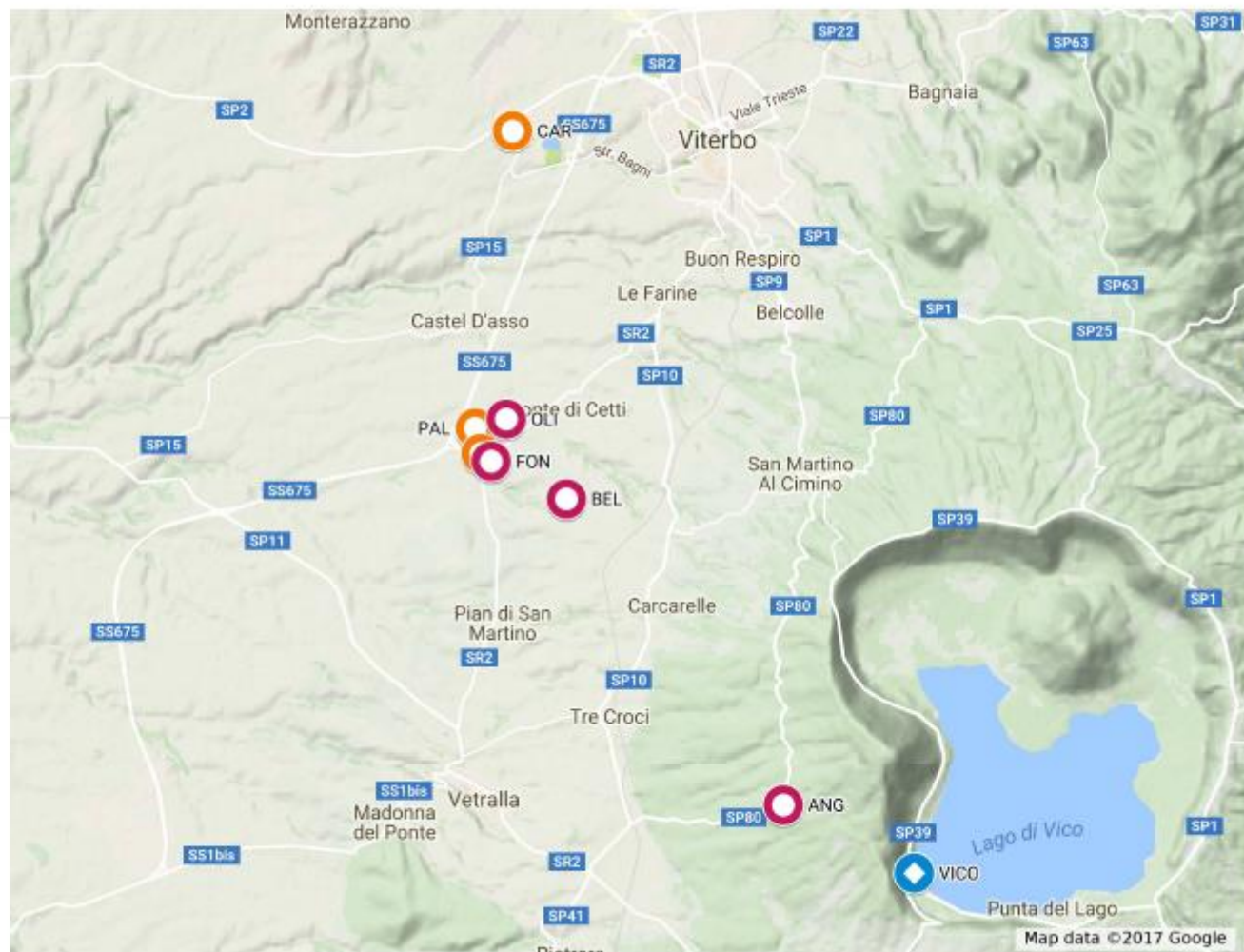

Supplement: Supplementary file 6 [file Image_1.pdf]
